# The New Age of Sudomotor Function Testing: A Sensitive and Specific Biomarker for Diagnosis, Estimation of Severity, Monitoring Progression, and Regression in Response to Intervention

**DOI:** 10.3389/fendo.2015.00094

**Published:** 2015-06-11

**Authors:** Aaron I. Vinik, Marie-Laure Nevoret, Carolina Casellini

**Affiliations:** ^1^Division of Endocrinology and Metabolism, Department of Medicine, Strelitz Diabetes and Neuroendocrine Center, Eastern Virginia Medical School, Norfolk, VA, USA; ^2^Impeto Medical Inc., San Diego, CA, USA

**Keywords:** sudomotor, sudorimetry, small nerve fiber, peripheral neuropathy, autonomic neuropathy

## Abstract

Sudorimetry technology has evolved dramatically, as a rapid, non-invasive, robust, and accurate biomarker for small fibers that can easily be integrated into clinical practice. Though skin biopsy with quantitation of intraepidermal nerve fiber density is still currently recognized as the gold standard, sudorimetry may yield diagnostic information not only on autonomic dysfunction but also enhance the assessment of the small somatosensory nerves, disease detection, progression, and response to therapy. Sudorimetry can be assessed using Sudoscan™, which measures electrochemical skin conductance (ESC) of hands and feet. It is based on different electrochemical principles (reverse iontophoresis and chronoamperometry) to measure sudomotor function than prior technologies, affording it a much more practical and precise performance profile for routine clinical use with potential as a research tool. Small nerve fiber dysfunction has been found to occur early in metabolic syndrome and diabetes and may also be the only neurological manifestation in small fiber neuropathies, beneath the detection limits of traditional nerve function tests. Test results are robust, accomplished within minutes, require little technical training and no calculations, since established norms have been provided for the effects of age, gender, and ethnicity. Sudomotor testing has been greatly under-utilized in the past, restricted to specialized centers capable of handling the technically demanding and expensive technology. Yet, evaluation of autonomic and somatic nerve function has been shown to be one of the best estimates of cardiovascular risk. Evaluation of sweating has the appeal of quantifiable non-invasive determination of the integrity of the peripheral autonomic nervous system, and can now be accomplished rapidly at point of care clinics with the determination of ESC, allowing intervention for morbid complications prior to permanent structural nerve damage. We review here sudomotor function testing technology, the research evidence accumulated supporting the clinical utility of measuring ESC, the medical applications of sudorimetry now available to physicians with this device, and clinical vignettes illustrating its use in the clinical decision-making process.

Evaluation of autonomic and somatic nerve function has now been shown to be one of the most important predictors of cardiovascular risk (1–6). While there are bedside means of evaluation of the integrity of the somatic nervous system (NIS-LL (7, 8), UENS (9), and MNSI (10), these are imprecise and the use of gold standard measures such as nerve conduction studies are burdensome, time consuming, and do not capture small nerve fiber function (11–13). Clearly a simple, rapid, and precise means of quantification of both large and small fiber function would greatly enhance the capacity to capture all the elements of peripheral and autonomic neuropathy. Autonomic function tests have been extraordinarily useful in diagnosing, treating, and better understanding of the complex mechanisms behind disturbances in balance of the function of the two domains of the autonomic nervous system, namely sympathetic and parasympathetic (14). The ability to capture time and frequency domains of heart rate variability (14), as well as baroreceptor sensitivity, has facilitated the evaluation of balance between the two arms of the autonomic nervous system, providing information on functional changes prior to the development of permanent structural damage and allowing the use of rebalancing therapeutic options (15). Small unmyelinated C and Aδ fibers in the periphery subserve somatic and autonomic nerve functions such as warm, cold, and pain perception, as well as innervating sweat glands. The gold standard for their evaluation has been skin biopsy and quantification of intraepidermal nerve fiber density, an invasive procedure (16, 17). On the other hand, the evaluation of sweating has the appeal of quantifiable non-invasive determination of the integrity of the peripheral autonomic nervous system. However, activation of sweat glands is complex and both sympathetic and parasympathetic innervation contribute to normal sweat gland function (18). A variety of techniques have capitalized on quantifying sweating (19) or the innervation of sweat glands (20, 21) to capture the impact of disordered regulation on structure and function of sweat glands as surrogates for the underlying somatic and autonomic dysfunction.

Sudorimetry – or sudomotor function testing – the science of measuring the function of sweat gland innervation, is unique among the autonomic function tests in that it evaluates the peripheral sympathetic system but relies principally on cholinergic post-ganglionic neurotransmission. Recently, a newer sudomotor function technology (Sudoscan) has become available and adds a new tool to test at point of care in the clinic when autonomic testing had been previously restricted to specialized neurological laboratories. With an aging, increasingly obese population with diabetes and prediabetes, disorders that manifest autonomic dysfunctions are becoming common and sudorimetry may allow earlier intervention for these morbid complications.

## Sudomotor Function Technology: The Why and the How

**Figure 1:**
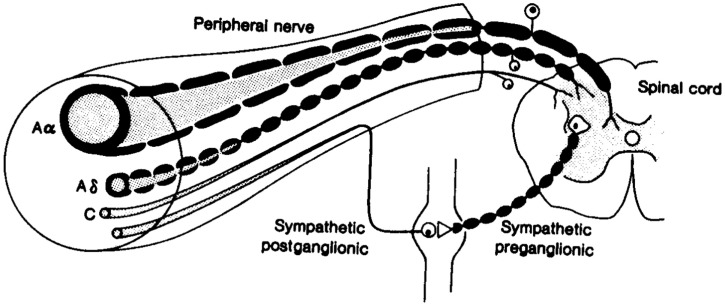
**Fibers of the peripheral nervous system: note the afferent and efferent connections among the small Aδ and C fibers (71)**.

Sweat glands being present at the skin surface, their stimulation and measured response have been extensively utilized to assess disorders of the autonomic system non-invasively, specifically the peripheral sympathetic system. Sudomotor nerves are C-fibers, thin unmyelinated, or poorly myelinated with primarily cholinergic neurotransmission, wherein the neurotransmitter at the ganglion is acetylcholine, the principal neurotransmitter of the parasympathetic nervous system. However, epinephrine, norepinephrine, vasoactive intestinal peptide (VIP), atrial natriuretic peptide, calcitonin gene related polypeptide (CGRP), galanin, ATP, and substance P have also been localized to peri-glandular nerves (22). The nerve fibers are structurally similar to small nerve fibers of warm and cold thermal perception, and hence prone to damage by the same metabolic insults (Figure 1).

Small nerve fiber dysfunction has been found to occur early in metabolic syndrome and diabetes (23, 24), and may also be the only neurological manifestation in small fiber neuropathies (25, 26). Apart from skin biopsy, measuring small nerve fiber function has been limited to assessment of symptoms and quantitative sensory testing for warm, cold, and pain thresholds, which lack the precision required for a practical clinical profile or use in the research arena.

The unique qualities of sudomotor function tests are that, in clinical application, they may yield diagnostic information not only of autonomic dysfunction but also enhance the assessment of the small somatosensory nerves (27, 28). To date, however, most sudomotor function testing procedures were not accessible for widespread use but rather confined to specialized autonomic labs. Tests using iodine and starch (thermoregulatory sweat test), the Neuropad (29, 30), or iontophoretic stimulation and quantification of sweating are either time consuming, technically demanding, or lack sensitivity, specificity, or reproducibility to be of practical use (31–33). Furthermore, they were prone to both environmental and patient-specific confounders like room temperature, medications, food, and habituation. Excellent reviews on sudomotor function tests can be read elsewhere (19).

In contrast, Sudoscan™ is based on different electrochemical principles (reverse iontophoresis and chronoamperometry) to measure sudomotor function than prior technologies, affording it a much more practical and precise performance profile for routine clinical use with potential as a research tool. The device consists of a simple desktop computer connected to two sets of large surface stainless steel electrodes: two for application of the palms, and two for the soles. The patient places both hands and feet simultaneously on the designated electrodes and a painless scanning process ensues over the course of 2–3 min. A low DC voltage is incrementally applied to the electrodes, ranging from 1 to 4 V, with the left and right electrodes serving alternatively as cathode and anode. At voltages under 10 V, the thick stratum corneum is electrically insulating; the sweat glands, however, consist of a cellular bilayer and therefore can transmit electrically charged ions to the skin surface and the electrodes (reverse iontophoresis). The current of sweat chloride ions generated in response to the incremental voltage applied can be quantified and reflects the function of the sweat gland, and hence its C fiber innervation. This chloride ion current is reported as electrochemical skin conductance (ESC) – the inverse of resistance – measured in microSiemens (μS).

The palms and soles are specifically assessed due to their very high density of sweat glands and the frequency of small nerve degeneration occurring in a length-dependent fashion; i.e., affecting the distal-most nerve endings first. Also, by alternating the left and right extremities as anode and cathode, each extremity is evaluated separately during the 3 min scan, and asymmetry between the extremities can be calculated by the device.

## Clinical Diagnostic Evaluation Using ESC: Assessment of Somatic and Autonomic Nerve Dysfunction

The clinical diagnostic accuracy of Sudoscan™ has been evaluated for diabetic peripheral sensorimotor polyneuropathy (DPN), peripheral small fiber neuropathy, and autonomic neuropathy against other standard methods.

**Figure 2:**
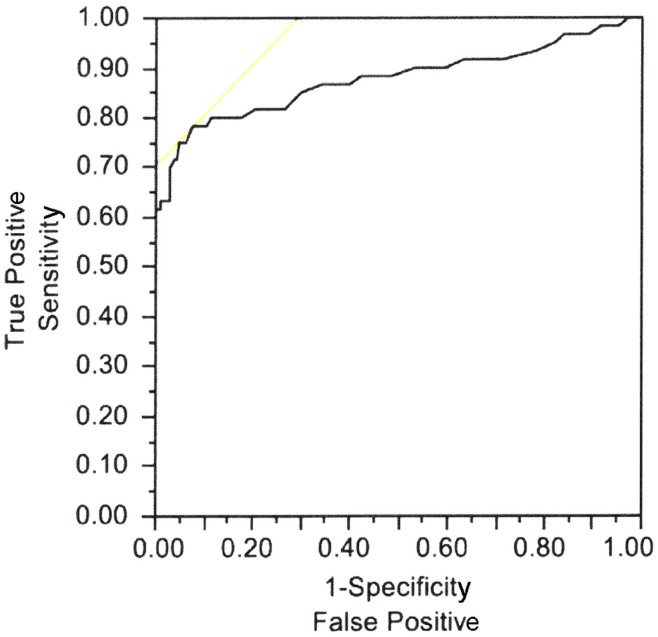
**Electrochemical skin conductance of feet receiver–operator characteristic (ROC) curve to reflect diabetic neuropathy**. Area under the curve = 0.8755, *p* < 0.0001 (34).

Casellini et al. (34) examined 83 type 1 and 2 diabetic patients, both with and without DPN, and compared them to 210 healthy controls (HC). Diabetic patients with DPN had significantly worse ESCs of both feet and hands than patients without DPN and HC [56.3 ± 3 vs. 75.9 ± 5.5 and 84.4 ± 0.9 (*p* < 0.0001) for feet and 51.9 ± 2.4 vs. 67.5 ± 4.3 and 73.1 ± 0.8 (*p* < 0.0001) for hands]. Somewhat surprisingly, ESCs correlated significantly with clinical neuropathy scores of sensory, motor, and reflex function; somatic (quantitative sensory testing and nerve conduction studies) and autonomic (quantitative autonomic function testing) measures of DPN suggesting that the measure captures both somatic and autonomic nerve function. Using a Neuropathy Impairment Score of the Lower Limb (NIS-LL) >2 as the gold standard for the diagnosis of neuropathy, the sensitivity and specificity of ESC for the diagnosis of DPN were 78 and 92%, respectively, for feet, with an ROC area under the curve of 0.8755 (*p* < 0.0001) (Figure 2). Table 1 compares ESC results to vibration detection threshold (VDT) and sural amplitude and suggests that Sudoscan is a moderately sensitive but highly specific tool to detect DPN. The investigators further determined the correlations between sudorimetry and somatic and autonomic function as shown in 48 type 2 diabetic patients (Table 2). There were strong correlations between ESC and both somatic (sensory and motor) and autonomic functions; sudorimetry scores were also able to separate painful from non-painful DPN.

**Table 1:** **Diagnostic efficiency of feet and hands electrochemical skin conductance to reflect diabetic neuropathy (Neurologic Impairment Score – lower limbs) (34)**.

	Criterion^a^	Sensitivity	Specificity	+LR	−LR	+PV	−PV
Hands ESC	64	78.33	85.71	5.48	0.25	61.04	93.26
Feet ESC	77	78.34	92.38	10.28	0.23	74.6	93.72
Total NIS-LL	1.5	76.67	85.71	5.37	0.27	95.83	46.15

*^a^Criterion corresponding with highest Youden index*.

*ESC, electrochemical skin conductance; +LR, positive likelihood ratio; −LR, negative likelihood ratio; NIS–LL, Neurologic Impairment Score–Lower Legs; +PV, positive predictive value; −PV, negative predictive value*.

**Table 2:** **Spearman’s ρ ranked correlations of feet ESC with clinical, somatic, and autonomic measures of neuropathy in 48 patients with type 2 diabetes (34)**.

	Feet ESC	Spearman ρ	*p*-Value^#^
**CLINICAL NEUROPATHY SCORES**			
DM duration	Feet ESC	−0.4904	0.0005
Neurologic symptom score (NSS)	Feet ESC	−0.4437	0.0016
Sensory score	Feet ESC	−0.6313	<0.0001
Motor score	Feet ESC	−0.5499	<0.0001
Total neuropathy score (TNS)	Feet ESC	−0.5851	<0.0001
Total NIS-LL motor	Feet ESC	−0.6687	<0.0001
Total NIS-LL sensory	Feet ESC	−0.6672	<0.0001
Total NIS-LL score	Feet ESC	−0.6160	<0.0001
**AUTONOMIC FUNCTION MEASURES (QAFTs)^a^**			
Expiration/inspiration ratio (E/I)	Feet ESC	0.4858	0.0005
Valsalva ratio	Feet ESC	0.3182	0.0275
Deep breathing total spectral power (TSP)	Feet ESC	0.4745	0.0008
Deep breathing sdNN	Feet ESC	0.4252	0.0029
Deep breathing rmsSD	Feet ESC	0.3778	0.0088
Valsalva TSP	Feet ESC	0.4320	0.0024
Valsalva sdNN	Feet ESC	0.3708	0.0103
Valsalva rmsSD	Feet ESC	0.3026	0.0387
**QUANTITATIVE SENSORY TESTING (QSTs)**			
Great toe vibration detection	Feet ESC	−0.5003	0.0004
Great toe pressure (monofilament)	Feet ESC	−0.6250	<0.0001
Great toe cold perception	Feet ESC	−0.4625	0.0012
Great toe warm perception	Feet ESC	−0.4857	0.0006
**PAIN SCORES (NRS)**			
Great toe symptomatic pain	Feet ESC	−0.4397	0.0022
Great toe mechanical pain	Feet ESC	−0.3508	0.0168
**NERVE CONDUCTION STUDIES (NCS)**			
Peroneal ankle amplitude (Amp)	Feet ESC	0.4889	0.0005
Peroneal below fibula Amp	Feet ESC	0.5867	<0.0001
Peroneal below fibula conduction velocity (CV)	Feet ESC	0.4724	0.0008
Peroneal above fibula Amp	Feet ESC	0.5496	<0.0001
Peroneal above fibula CV	Feet ESC	0.4381	0.0021
Sural Amp	Feet ESC	0.5234	0.0043
Sural CV	Feet ESC	0.5503	0.0024
**LABORATORY VARIABLES**			
Urine albumin/creatinine ratio	Feet ESC	−0.3724	0.0196

*^a^Data are log-transformed*.

*^#^Spearman’s ρ rank test was applied*.

*DM, diabetes mellitus; ESC, electrochemical skin conductance; NIS-LL, neuropathy impairment score of lower legs; NRS, numeric rating score; rmsSD, root mean square of the difference of successive *R*–*R* intervals; sdnn, sample difference of the beat to beat (NN) variability*.

In this cohort, sweat gland size and morphology were not available. There is evidence that sweat glands and sweat gland duct diameter may be smaller in patients with DPN (35, 36); one likely etiology is the thickened endothelial basement membranes of feeding capillaries resulting in microvascular damage of cutaneous structures. However, the contribution of these morphological abnormalities to diabetic sudomotor dysfunction is not known.

**Figure 3:**
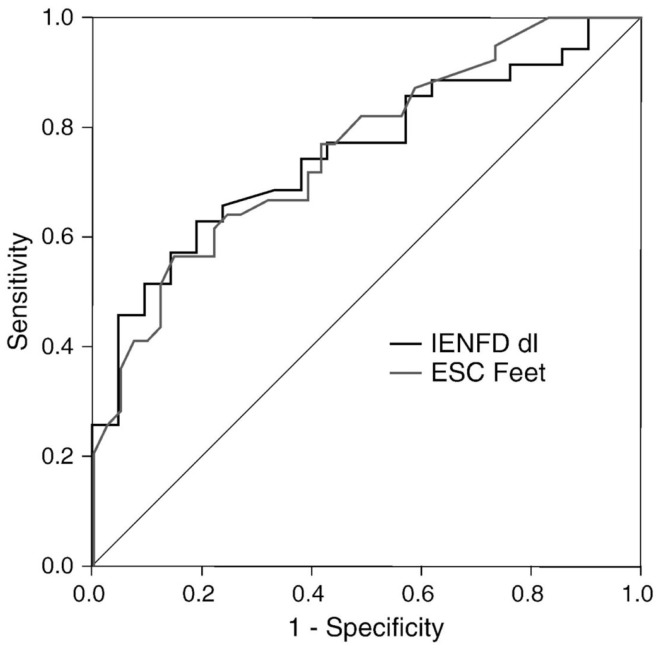
**Receiver operating characteristics (ROC) curves for feet ESC and skin biopsy using abnormal Utah Early Neuropathy Score (UENS) as the gold standard**. Area under the curve (AUC) for ESC feet and intraepidermal nerve fiber density (IENFD) were similar (0.761, 0.752, respectively) (9).

Distal symmetric polyneuropathy (DSP) presents with small fiber-predominant dysfunction; early detection currently relies on skin biopsy with quantification of intraepidermal nerve fiber density (IENFD). Smith et al. (9) studied 55 patients with suspected DSP (idiopathic or diabetes-related) and 42 HC to evaluate the diagnostic performance of ESC. Using an abnormal Utah Early Neuropathy Score (UENS) ≥4 as the diagnostic gold standard, feet and hands ESC were significantly lower in DSP subjects than HC, regardless of the etiology of DSP: 64 ± 22 vs. 76 ± 14 μS, and 58 ± 19 vs. 66 ± 18 μS, respectively. More importantly, ESC and IENFD had similar diagnostic performance in detecting DSP, with a ROC area under the curve of 0.761 and 0.752, respectively (Figure 3). Mirroring the findings of Casellini et al., feet ESC had a very high negative predictive value (83%) and significantly correlated with both symptoms and signs assessed using the Michigan Neuropathy Screening Instrument (MNSI) and the Utah early neuropathy symptom scale(UENS). Also, quantitative sudomotor axon reflex testing (QSART) at the foot and feet ESC were correlated, but only moderately (0.348, *p* < 0.015); the authors attributed this observation to the large standard deviation in the QSART data, a reflection of variability in sweat volume produced. An alternate explanation is that Cl ion transport may reflect functions other than sweating alone (9).

Yajnik and colleagues (37) investigated DPN and autonomic neuropathy in 265 type 2 diabetic patients using ESC measurements, MNSI, and vibration perception threshold (VPT). Again, feet ESC were significantly correlated with MNSI Part A (symptoms, *p* < 0.05) and Part B (physical examination, *p* < 0.001), and VPT (*p* < 0.001). Gin et al. (38) similarly compared ESC to VPT in the diagnosis of DPN among 142 diabetic (67% type 2) individuals. The correlation coefficient between foot ESC and VPT was −0.45 (*p* < 0.0001). VPT and ESC also correlated with retinopathy status [*r* = 0.52 (SD 0.07) and −0.52 (SD 0.07), respectively; *p* < 0.0001] and with creatinine clearance (*p* = 0.003) in the Modification of Diet in Renal Disease (MDRD).

As in the study by Casellini and collaborators, a number of projects have shown that ESC measurement may be a simple tool for early identification of autonomic neuropathy, and may be useful in screening for sub-clinical cardiac autonomic neuropathy (CAN). Calvet et al. (39) in 232 diabetic individuals showed a high correlation between CAN risk score (calculated from ESC) and the low frequency (LF) component, a measure of sympathetic and baroreceptor function (40), during moderate activity (*r* = 0.47, *p* < 0.001). When taking the LF power component during moderate activity <90 ms^2^ as a reference value, the ROC area under the curve for the CAN risk score was 0.77, with a sensitivity and specificity of 83 and 63%, respectively.

Below are summarized some of the major studies completed using ESC measurement in a clinical diagnostic capacity (Table 3).

**Table 3:** **Feet ESC diagnostic accuracy vs. established clinical tools**.

Study	Diagnostic variable	Comparison	Sensitivity (%)	Specificity (%)	PPV (%)	NPV (%)
Casellini (34)	DPN	NIS-LL	78	92	74	93
Smith (9)	Distal symmetric polyneuropathy	UENS	77	67	59	83
Yajnik (37)	DPN	VPT	73	62	N/A	N/A
Eranki (41)	DPN	VPT	82	55	N/A	N/A
Calvet (39)	CAN – diabetes	CARTs	83	63	N/A	N/A
Ozaki (42)	Diabetic kidney disease	eGFR, ACR	94	78	81	93

*ACR, albumin creatinine ratio; CAN, cardiac autonomic neuropathy; CARTs, cardiac autonomic reflex tests; DPN, diabetic peripheral neuropathy; eGFR, estimated glomerular filtration rate; NIS-LL, Neuropathy Impairment Score – Lower Limbs; PPV, positive predictive value; NPV, negative predictive value; UENS, Utah Early Neuropathy Score; VPT, vibration perception threshold*.

## Validation of ESC Robustness

For ESC measurement to be clinically meaningful, widely applicable thresholds for normal and abnormal sudomotor function had to be determined, and high reproducibility of results had to be demonstrated. One of the weaknesses of many sudomotor function tests has been their wide range of normal values (QSART) and their sensitivity to temperature, age, medications, and food. Habituation (decreases in result amplitude with repeated testing) is a particular problem with sympathetic skin response.

Initially, a large cohort of healthy subjects (432 females, 177 males; age >20-years-old; without known neuropathy, diabetes, or chronic medical condition; BMI < 25) were tested using Sudoscan™ to determine population normative values. The results of this study are depicted in Table 4 and indicate the following: (1) there is almost no drop-off in normal sudomotor function from age 21 to 80; (2) results are not significantly different between the sexes; and (3) a threshold of 60 μS for hands and feet does not result in a significant number of false positives (healthy subjects being diagnosed with dysautonomia). Only in the eighth decade, do mean ESC values decrease slightly (eighth vs. third decade feet ESC: 75.5 ± 9.3 vs. 83.9 ± 5.8 μS for women, respectively; 76.0 ± 9.4 vs. 81.7 ± 7.6 μS for men, respectively).

**Table 4:** **Effects of age and gender on ESC measurements in 609 normal subjects**.

	Age (years)^a^						
	Overall	21–30	31–40	41–50	51–60	61–70	71–80
**FEMALE**							
**ESC hands**	(*n* = 432)	(*n* = 29)	(*n* = 73)	(*n* = 136)	(*n* = 143)	(*n* = 28)	(*n* = 23)
Mean ± SD	74.0 ± 8.7	74.7 ± 9.6	75.9 ± 8.8	74.0 ± 8.4	73.8 ± 8.3	71.1 ± 10.1	71.0 ± 9.6
Median	75.0	75.0	77.0	74.5	75.0	70.3	71.5
80th percentile interval	61.5–85.0	62.6–86.2	62.6–87.0	62.0–85.0	62.0–85.0	58.9–83.6	55.9–81.4
**ESC feet**
Mean ± SD	82.8 ± 5.8	83.9 ± 5.8	84.2 ± 4.8	83.1 ± 5.2	82.7 ± 5.6	82.2 ± 4.2	75.5 ± 9.3
Median	83.5	84.5	85.0	84.5	83.0	83.0	79.0
80th percentile interval	76.0–89.0	79.1–90.0	77.1–89.5	76.5–89.0	75.5–89.5	76.7–86.5	65.1–86.7
**MALE**							
**ESC hands**	(*n* = 177)	(*n* = 46)	(*n* = 30)	(*n* = 33)	(*n* = 42)	(*n* = 10)	(*n* = 16)
Mean ± SD	74.9 ± 9.6	76.0 ± 10.1	77.4 ± 6.6	75.2 ± 10.7	75.8 ± 7.7	72.3 ± 11.6	66.2 ± 10.0
Median	76.5	77.5	78.3	78.0	76.3	75.0	68.0
80th percentile interval	62.5–86.5	63.0–87.5	69.1–85.6	61.1–87.9	66.7–86.4	56.1–85.6	52.5–78.3
**ESC feet**
Mean ± SD	81.5 ± 7.0	81.7 ± 7.6	81.2 ± 5.6	81.0 ± 7.1	84.0 ± 5.7	81.5 ± 4.4	76.0 ± 9.4
Median	82.5	82.5	81.0	83.0	84.5	81.8	79.3
80th percentile interval	73.0–89.5	73.8–90.5	75.0–88.2	71.3–89.3	76.1–90.0	76.7–86.2	67.5–83.5

*^a^Internal data provided by Impeto Medical, Inc*.

Cohorts of diabetic patients, both with and without DPN, then underwent ESC testing by Gin and Yajnik (37, 38); these studies indicated that a threshold of 60 μS clearly separated normal (“green” zone) from abnormal (“yellow” and “red” zones) sudomotor function for both the hands and feet. A second threshold of 40 μS denoted the level below which sudomotor function was severely impaired (“red” zone) and correlated closely with advanced peripheral neuropathy and attendant complications, e.g., foot ulceration (34, 37, 38).

Three critical studies were then completed in the US (9, 34, 43) and led to further refinement of ESC thresholds by comparing ESC scores against multiple validated DPN tools. Results of these studies demonstrated that: (1) the initial 40 and 60 μS thresholds best applied to Asian populations; (2) optimal sensitivity of ESC testing in Caucasians required raising the foot ESC threshold to 70 μS; and (3) African Americans had unique sudomotor physiology requiring specific ESC thresholds for both hands and feet. Though threshold refinement by ethnic background has not yet been clinically validated for all populations, using the adjustments depicted in Table 5 below is already allowing much stronger accuracy of ESC result interpretation and clinically meaningful information for the physician.

**Table 5:** **Electrochemical skin conductance (ESC) zones for normal sudomotor function (green), moderate sudomotor dysfunction (yellow), and severe sudomotor dysfunction (red) as defined by racial background**.

Race	Hands ESC red zone	Hands ESC yellow zone	Hands ESC green zone	redFeet ESC red zone	Feet ESC yellow zone	Feet ESC green zone
Caucasian	0–40	40–60	60–100	0–50	50–70	70–100
African-American Ancestry	0–30	30–50	50–100	0–40	40–60	60–100
Asian, Indian	0–40	40–60	60–100	0–40	40–60	60–100

*All numeric values in micoSiemens (μS)*.

*Data from Casellini, Gordon Smith, and Freedman (9, 34, 43)*.

Reproducibility testing was performed under various stress scenarios as well as normal conditions.

### Effect of chronic disease

Electrochemical skin conductance measurements were assessed twice on the same day in patients with at least one cardiovascular risk and in patients with diabetes. Results were compared using a Bland–Altman plot. The coefficient of variation was 7% in hands and 5% in feet in patients with cardiovascular risk and 15% in hands and 7% in feet in patients with diabetes in whom the coefficient of variation for glycemia between the two measurements was 32%.

### Effect of glycaemia

As this technology is frequently applied to pre-diabetic or diabetic patients with potentially high glycemic variations, ESC measurements were performed twice in 12 patients, once when blood glucose levels were ≥325 mg/dL and once when normoglycemic. The coefficient of variation between the two sets of measurements from a Bland–Altman plot was 10% in the feet. This is not unexpected as hyperglycemia induces a state of hyperosmotic dehydration. In hyperosmolar hyperglycemic non-ketotic syndrome, there is an absence of sweating in an attempt to conserve water, probably with diversion of blood from cutaneous microvascular beds. It may be that the ESC test, despite supra-physiological stimulation of the sweat glands, is unable to overcome the body’s conservation mechanism, leading to changes in ESC results during hyperglycemia. Just as the stomach is paralyzed by hyperglycemia and gastric emptying should only be measured when blood glucose levels are near normal (<250 mg/dL) so should sudorimetry strive to be examined only when the blood glucose is near normal.

### Effect of exercise

As exercise could influence sweat function, measurements were taken before and after an ergonomic bicycle exercise test at a level of 87% of maximum heart rate. ESC before and after measurements had a coefficient of variation of 4% in the feet and 8% in the hands.

### Effect of the device

Three measurements were performed in 21 patients using three different devices. There was no significant difference between the three measurements, and the paired Spearman test evidenced a coefficient of correlation higher than 0.96 for each comparison (*p* < 0.0001) (44).

## Sudomotor Function and Aging

It is now well accepted that important changes to the autonomic nervous system occur with aging, leading not only to increased morbidity and mortality but also complicating the therapeutic care of comorbidities of elderly patients, e.g., the use of sympatholytic drugs and diuretics for hypertension or the optimization of glucose control for diabetes (45). Extensive research indicates that both sympathetic and parasympathetic dysfunction occur with age, with tonic vagal modulation decreasing and a relative or absolute increase in sympathetic tone (46, 47). Schmidt notes that neurons in the aging sympathetic ganglia are mostly intact, whereas dendrites, axons, and synapses can be severely abnormal and very likely contribute to faulty signaling (45).

It must be noted, however, that the degree of autonomic dysfunction in the elderly can be highly dependent on fitness level, obesity, and to a lesser extent gender (48–50). Our data on ESC only show a reduction in this function in the 8th decade (Table 4).

Some studies have indicated decreased sweat volumes on the dorsum of the hands and feet with aging (51), while Low found significant decreases in quantitative sudomotor axon reflex test (QSART) only in the lower extremities (52). In an earlier publication, Low had saliently concluded that age-related changes of the ANS vary with “the system tested, the site examined, and the particular test used” (53). We contend that ESC reflects not only sweating but somatic neurologic function which declines with age (54).

Sudoscan™ examines the palms and soles, and measures an electrochemical response from the skin rather than a sweat volume or latency of response. As such, ESC results across ages 21–80 in healthy individuals have been found to remain fairly constant, and certainly permit use of a uniform set of diagnostic thresholds. This in turn allows identification of sudomotor dysfunction in older adults equally as easily as in younger ones (Table 4).

## ESC as a Tool to Measure Progression and Regression of Disease

One of the most difficult and frustrating tasks in peripheral neuropathy has been to measure disease progression and response to therapeutic intervention with an easy, objective, and accurate tool. Knowing that salvaging large nerve fibers is practically impossible, the Holy Grail is to detect early nerve damage – perhaps before metabolic parameters indicate any disease – and measure response or lack thereof accurately and quickly, allowing for treatment modification. Several research studies have now demonstrated that ESC may be a clinically meaningful tool in measuring neuropathy progression and regression.

In a cohort of 52 type 1 and 115 type 2 diabetic patients followed for 1 year, Calvet and colleagues (39) showed that a decrease in hand and foot ESC was observed in Type 2 patients treated without insulin, while a significant increase was observed in patients receiving insulin (−3.8 ± 9.7 vs. 1.0 ± 9.7 μS, *p* = 0.02 for the hands and −2.2 ± 7.5 vs. 4.1 ± 8.8 μS, *p* < 0.001 for the feet). Type 1 patients, all on insulin, had a non-significant increase in their feet ESC as well (1.0 ± 7.2 μS). During this period, none of the cohorts (insulin/no insulin) had any significant change in their glycemic control which would signal a need for therapeutic adjustment (delta HbA1c 0.07 ± 0.60% and 0.13 ± 0.87%, respectively); yet, clearly the no insulin group was experiencing ongoing peripheral nerve loss. This lack of relationship with glycemic control argues in favor of an effect of insulin *per se* on Cl^−^ ion transport. Insulin is known to enhance the activity of Na^+^–K^+^–2Cl^−^co-transporter (NKCC). Recently, Sun and colleagues demonstrated that insulin treatment remarkably enhanced the forskolin-stimulated Cl^−^secretion in epithelial A6 cells, which was associated with an increase in apical Cl^−^ conductance by upregulating mRNA expression of both CFTR and NKCC (55). It would be of interest to examine the direct effects of euglycemic insulin administration as well as insulinotropic drugs on ESC quantification of Cl transport. Furthermore, sudorimetry may well prove useful in the evaluation of hyperinsulinemic insulin resistant states.

Conversely, Raisanen and colleagues (56) demonstrated that therapeutic intervention could improve small fiber function, and is easily measured using ESC. Residents of Tornio, Finland, who were at high cardiometabolic risk (BMI >35 kg/m^2^, very low VO_2_max, diabetes, or heart disease), completed a supervised health promotion program for 12 months. Among 154 female participants with the lowest fitness level at baseline, those performing the highest level of weekly activity showed the greatest improvement in ESC scores, which was more pronounced than the changes in weight, waist circumference, or VO_2_max (Table 6). Raisanen’s results confirm previous findings by Smith et al. (57) that small C-fibers damaged by metabolic stress can recover from simple lifestyle interventions lending further support to the notion that sudorimetry detects small somatic nerve fiber function that do not necessarily innervate sweat glands.

**Table 6:** **Change between baseline and 12-month follow-up in weight, waist, VO_2_max, and ESC risk score value in 154 women included in a lifestyle intervention program (56)**.

	Without follow-up of training level (*n* = 72)		Low weekly activity^a^ (*n* = 62)		High weekly activity^b^ (*n* = 20)		*p*
	Mean	SD	Mean	SD	Mean	SD	
Change in weight (kg)	−0.9	3.4	−1.6	4.0	−3.3	4.8	NS
Change in waist (cm)	−2.1	4.7	−2.4	4.5	−3.6	5.9	NS
Change in estimated VO_2_max (METs)	+0.5	0.9	+0.8	0.9	+1.1	1.2	NS
Change in hand ESC (μS)	+5.0	8.4	+3.0	9.4	+8.4	12.3	0.043
Change in foot ESC (μS)	+5.6	8.9	+4.9	8.9	+10.8	12.8	0.024
Change in ESC risk score (%)	−5.1	5.3	−4.7	6.4	−8.5	6.8	0.027

*^a^Less than 150 min of moderate activity and 75 min of high activity*.

*^b^More than 150 min of moderate activity or 75 min of high activity; moderate activity 3–7 METs; high activity >7 METs*.

These studies indicate that ESC may provide information on the metabolic health of a patient, in particular on their small nerves, which may not otherwise be known to or measurable by the physician. Initial ESC results may be used to alter management and repeat testing after intervention may accurately measure whether the treatment is effective or if further changes are necessary. Significantly, the test is easy and precise enough to be clinically useful and meaningful for everyday outpatient practices.

One often neglected aspect of peripheral neuropathy is its impact on the patient’s quality of life. Excellent tools such as the Norfolk Quality of Life-Diabetic Neuropathy (QOL-DN) have been developed to measure the patient’s experience of diabetic neuropathy. The nerve fiber-specific domains of QOL-DN, in particular, have been shown to correlate with objective measures of nerve function (58–60). QOL-DN has also been shown to be a reliable indicator of the impact on QOL of symptomatic peripheral neuropathy experienced by patients with V30M Transthyretin Familial Amyloid Polyneuropathy (TTR-FAP) (61) In the Tafamadis study, Coelho and colleagues (62, 63) demonstrated that treatment of patients with TTR-FAP was associated with an improvement of Norfolk QOL scores of symptoms and activities of daily living in parallel with improved NIS-LL, supporting the notion that the QOL tool can be nerve fiber specific. Concurrently, early studies have shown that ESC is able to clearly discriminate the degree of small nerve fiber dysfunction in symptomatic vs. asymptomatic TTR-FAP (mean feet ESC: 42 ± 27 vs. 81 ± 10 μS; *p* < 0.0001, respectively) (unpublished data). The next obvious step would be to measure the impact that ESC and QOL-DN together could have on disease staging and management in FAP, diabetes mellitus, and other small fiber neuropathies.

## Conclusion

Small nerve fibers are starting to be recognized as valuable neuronal structures in the investigation of neuropathic diseases. They are thin, poorly myelinated, or unmyelinated, and therefore prone to destruction early in many pathological processes and often symptomatically debilitating. Indeed, the earliest changes appear to occur in the lower back, forearm, and thenar eminence as shown using Contact Heat Evoked Potential (CHEPS) (64), challenging the dogma that neuropathy is a distal to proximal disorder. Small nerves can be assessed objectively, and recognition of early dysfunction can allow intervention, treatment, and potentially cure. Though skin biopsy with IENFD is currently recognized as the gold standard in the evaluation of small fibers, sudorimetry technology has evolved dramatically; appears to be a rapid, non-invasive, robust, and accurate biomarker for small fibers; and can easily be integrated into clinical practice. Disease detection, progression, and response to therapy have been shown to be measurable with ESC, and it may eventually assist the physician in monitoring more challenging disorders like CIDP. To allow for global evaluation of sudomotor function, however, the technology would need to be expanded in order to obtain information on dermatomes across the entire body.

## Case Vignettes on the Clinical Utility of Sudorimetry

Prior studies demonstrate that ESC technology is able to rapidly and precisely measure the effects of a therapeutic intervention; furthermore, individual patient examples are suggesting similar results. Below are two patients whose small nerve fiber function was uniquely measurable by sudorimetry.

### Vignette 1

**Figure 4:**
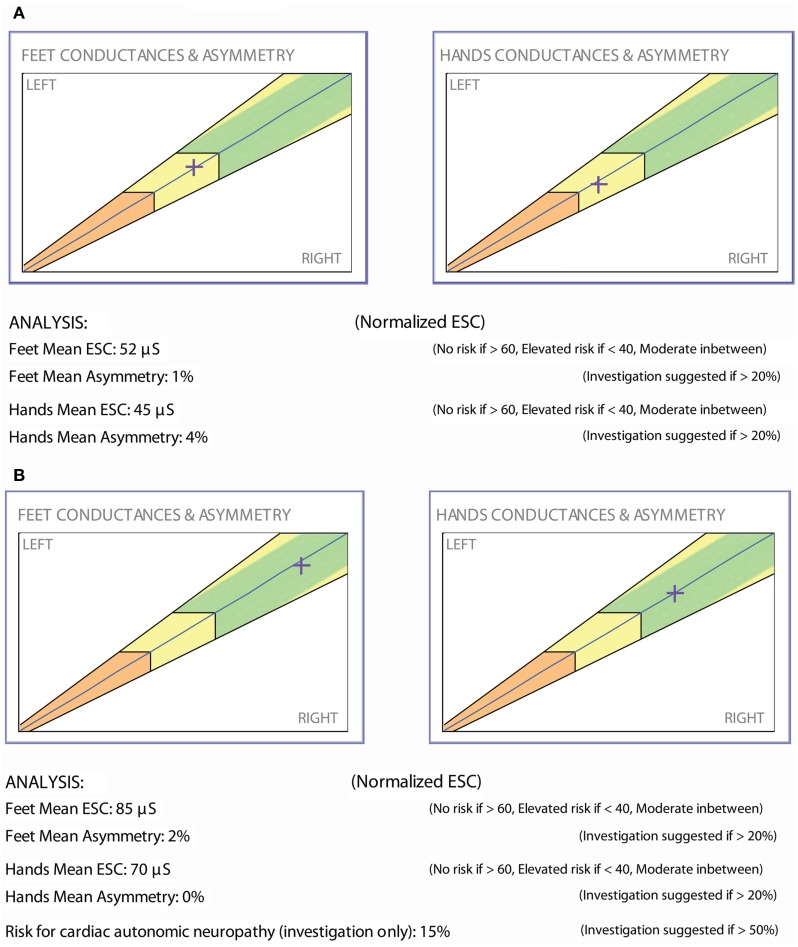
**(A)** ESC report dated October 19, 2012. **(B)** ESC report dated March 6, 2014.

The patient is a 47-year-old woman with well-controlled type 1 diabetes (HbA1c 6.4–7.5%) and 8+ years of painful diabetic neuropathy and gastroparesis. ESC measurements (Figure 4A) and cardiac autonomic functions were abnormal: heart rate variability response to expiration/inspiration (E/I), Valsalva maneuver, and the ratio of the RR interval for the 30th to the 15th beat upon standing were 1.05, 1.10, and 1.06, respectively. Gastroparesis was confirmed by a gastric emptying study and required a combination of psyllium fiber, metoclopramide, and erythromycin EES. Alpha lipoic acid (ALA) 600 mg po bid was prescribed. Ten months later, the patient returned with normalized ESC results (Figure 4B), complete resolution of the gastroparesis, but unchanged peripheral neuropathy symptoms, autonomic functions (E/I, Valsalva, and 30:15 ratios 1.10, 1.08, 1.08, respectively), and HbA1c.

Past research has suggested that ALA may benefit diabetic neuropathy, but objective quantification of its therapeutic effects has been difficult (65). The authors of a 2012 meta-analysis of ALA trials confirmed that 3 weeks of intravenous ALA results in significant and clinically relevant reductions in neuropathic pain (66) but declared that it was unclear if significant improvements measured for oral ALA were clinically relevant. Mijnhout et al., though, based their conclusions on the evolution of a single outcome measure, namely a ≥30% change in Total Symptom Score (TSS). The NATHAN 1 study, comparing 600 mg daily oral ALA vs. placebo, did not meet its primary endpoint (NIS-LL plus 7 neurophysiologic tests), despite 4 years of follow-up. However, Neuropathy Impairment Score, NIS-LL, and Neuropathy Symptoms and Change (NSC) score improved with ALA (67). NATHAN 1 firmly demonstrated that diabetic neuropathy, especially nerve conduction deficits, progresses quite slowly and that it may be more sensible to measure *improvements* in the treatment arm of a study rather than *deficit progression* in the placebo arm. ESC measurement was able to demonstrate improvements in small fibers with use of ALA in this patient; the ESC normalization correlated with resolution of her gastroparesis and opens the door to further investigate ESC’s utility in measuring pharmacological intervention in autonomic neuropathy.

### Vignette 2

**Figure 5:**
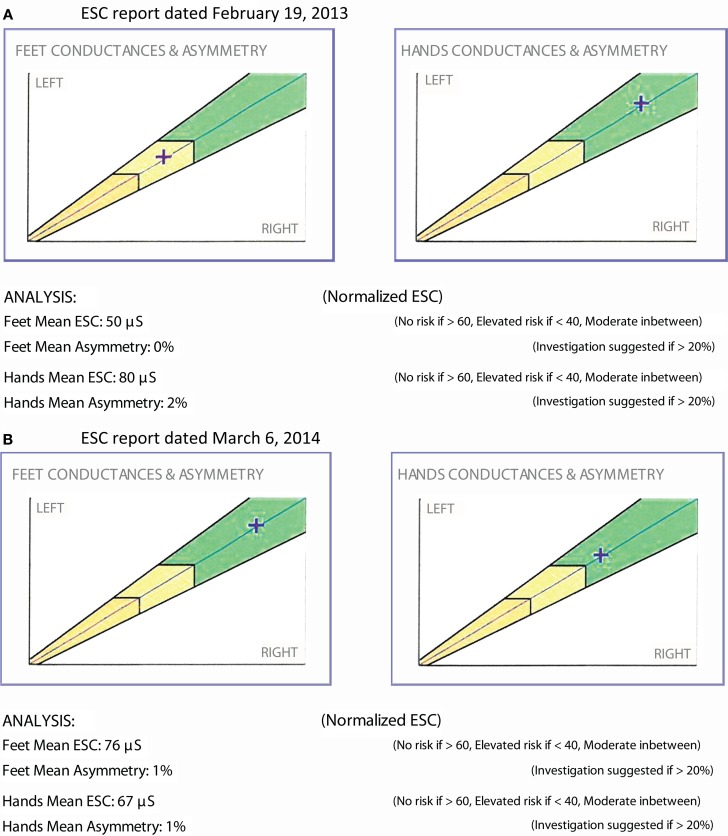
**(A)** ESC report dated February 19, 2013. **(B)** ESC report dated March 6, 2014.

The patient is a 50-year-old male with type 2 diabetes and a BMI of 30. He had been treated with metformin for several years and was optimally controlled. He denied any symptom of neuropathy; yet, ESC measurements demonstrated moderate sudomotor function loss (Figure 5A). A vitamin B12 level drawn that day was low (<150 pg/mL). He was started on vitamin B12 replacement and continued metformin. A repeat ESC 3 months later showed recovery of feet ESC scores (Figure 5B).

It has now been established that vitamin B12 threshold levels leading to impairment of nerve function is around 260 pmol/L (70 pg/ml) as shown in the Health ABC study (68). This case illustrates not only ESC’s ability to measure therapeutic response, but also its diagnostic capacity to detect a (possibly iatrogenic) neuropathy prior to symptomatology and identity candidates on metformin for B12 replacement. Fonseca and colleagues were unable to show that 24 weeks of treatment with a combination of methylcobalamin, methylfolate, and pyridoxal phosphate could alter vibration detection despite improving symptoms of neuropathy as well as quality of life (69, 70). Their study findings support the possible role of an objective measure like ESC in this clinical setting.

## Conflict of Interest Statement

The authors declare that the research was conducted in the absence of any commercial or financial relationships that could be construed as a potential conflict of interest.
